# Effects of Climate Change on Subterranean Termite Territory Size: A Simulation Study

**DOI:** 10.1673/031.011.8001

**Published:** 2011-07-01

**Authors:** Sang-Hee Lee, Tae-Soo Chon

**Affiliations:** ^1^ Division of Fusion and Convergence of Mathematical Sciences, National Institute for Mathematical Sciences, Daejeon, South Korea; ^2^ Department of Biological Sciences, Pusan National University, Busan (Pusan), South Korea

**Keywords:** insecticide, toxicity, Rajasthan, Gujarat

## Abstract

In order to study how climate change affects the territory size of subterranean termites, a lattice model was used to simulate the foraging territory of the Formosan subterranean termite, *Coptotermes formosanus* Shiraki (Isoptera: Rhinotermitidae), and the minimized local rules that are based on empirical data from the development of termites' foraging territory was applied. A landscape was generated by randomly assigning values ranging from 0.0 to 1.0 to each lattice site, which represented the spatially distributed property of the landscape. At the beginning of the simulation run, *N* territory seeds - one for each founding pair, were randomly distributed on the lattice space. The territories grew during the summer and shrank during the winter. In the model, the effects of climate change were demonstrated by changes in two variables: the period of the summer season, *T*, and the percentage of the remaining termite cells, σ, after the shrinkage. The territory size distribution was investigated in the size descending order for the values of *T* (= 10, 15, ... , 50) and σ (= 10, 15, ... , 50) at a steady state after a sufficiently long time period. The distribution was separated into two regions: the larger-sized territories and the smaller-sized territories. The slope, *m*, of the distribution of territory size on a semi-log scale for the larger-sized territories was maximal with T (45 ≤ *T* ≤ 50) in the maximal range and with σ in the optimal range (30 ≤ σ ≤ 40), regardless of the value of *N.* The results suggest that the climate change can influence the termite territory size distribution under the proper balance of *T* and σ in combination.

## Introduction

The recent climatic extremes, such as unseasonably high temperatures, due to global warming led to speculation that ecosystem disturbances are ubiquitous from local to global scales (Baes et al. 1977; Emanuel et al. 1980; Gardner et al. 1980; Schlamadinger et al. 1995). Climate change strongly affects ecosystem stability and resilience (Dukes and Mooney 1999; Dixon et al. 2003; Mendelsohn et al. 2006). For this reason, many researchers attempted to understand the effects in terms of system adaptability and variation at the ecosystem level (Pastor and Post 1988; Davis 1990; Bradley et al. 2005; Koneswaran and Nierenberg 2008). Climate changes also affect community and population dynamics within the ecosystem. For instance, the reduction of global Krill biomass is supposed to be strongly influenced by climate change (Hays et al. 2005). In the next century, Krill biomass across the Scotia Sea is expected to decrease by 95% (Murphy et al. 2007), threatening stabilization of aquatic ecosystems in the Southern Ocean food chain (Schmidt et al. 2003). As another example, global warming has been associated with the recent drastic population declines in pikas (or rock rabbits) (Huntly et al. 1986; Wei-Dong and Smith 2005), consequently contributing instability of community organization (Dearing 1997).

Beside the effect at the population and community levels, climate change may further affect animal behavior at the individual level. There have been numerous behavioral and physiological changes due to temperature increase (e.g. favorable conditions of slight temperature increase in insect pest development) (Cannon 1998; Bale et al 2002). Regarding behavior, weather-dependent foraging and breeding performance were observed in black kites in response to temperature change by Fabrizio (2003). Silva et al. (2007) presented that water temperature could be an effective factor in modulation of reproduction activities of pipefish, sexual recognition, mate preferences, and female-female interactions. Other research was also reported on behavioral changes along with physiological effects. Hazell et al. (2010) investigated thermal ecology and tolerance of aphids regarding impact of warming on winter limitation and suggested that heat coma may be a reliable indicator of fatal heat stress.

However, so far such studies mainly remain focused on terrestrial and aquatic systems, including total assemblages of plants and animals, but not subterranean animals because of the technical difficulties in observing animal behaviors and environmental factors in underground conditions. Understanding the effects of climate change on the subterranean ecosystem is indeed important because the terrestrial ecosystem is directly connected to the subterranean ecosystem (Wardle et al. 2004; Erb et al. 2008) and would be critical in maintaining sustainability both in natural (e.g., underground nests of animals) and human (e.g., house safety) systems.

In the present study, the effect of climate change on territory size of Formosan subterranean termites, *Coptotermes formosanus* Shiraki (Isoptera: Rhinotermitidae), was explored by using a lattice model proposed by Lee et al. (2007, 2008a), and an establishment of territories was investigated after a sufficiently long time. So far, the territorial behavior of *C. formosanus* has been extensively studied by mark-release-recapture methods to delineate the foraging ranges of colonies of the *C. formosanus* in the field (Su and Scheffrahn 1988a,b; Grace et al. 1992; Su et al. 1993; Messenger and Su 2005), which provided information to be used in termite control strategy and are related to the topic of territory size distribution. Territory size distribution is an important issue in ecological processes in social animals such as termites in subterranean conditions since the territory site reflects not only the optimal trade-off between the benefit (i.e. increased resources) and the cost (i.e. defense with increasing territory size), but also the foraging strategy of the termites (Lee et al. 2007).

## Model description and analysis

### Baseline model description

Termite foraging territory was simulated on a two-dimensional lattice space composed of cells of dimensions *L* × *L*, where *L* (= 200) is system size. Each cell was in one of three possible states: occupied by active termites (active termite cell), occupied by inactive termites (inactive termite cell), or empty (empty cell). The empirical analogy of an inactive termite cell is a tunnel tip surrounded by difficult-to-tunnel conditions such as physical obstacles and water barriers.

In the model, the landscape was produced by allotting randomly generated values, ranging from 0.0 to 1.0, to each cell. The value represented the degree of ease in constructing tunnels. Higher values corresponded to favorable soil conditions for easy tunneling. Thus, the value can be interpreted as a transition probability, *P_trans_,* for an active termite cell to grow into its neighboring cell.

In addition, the seasonal cycles of summer and winter were incorporated into the model construction. For the sake of theoretical simplification, it was assumed that seasons change according to a step function with two time scales: summer and winter. The simulated territory grew in the summer and shrank in the winter. In order to fit the time scale, the experimental data reported by Bess (1970) and Li et al. (1979) was compared. The iteration time *T* was defined as cycle duration presenting the perriod of termite activity matching to field conditions. The iteration time of *T* = 36 corresponds to the summer duration of New Orleans, Louisiana (Lee et al., 2007). Unlike the summer season, the winter season had no interactions among cells because real termite territory is shrunk without invasion into unexplored areas. For this reason, our territory model does not require iteration time to simulate the shrinkage process. Namely, the process in the winter season was fulfilled in one iteration time (*T* = 1). At the beginning of the simulation run, *N* active termite cells, representing the number of founding pairs, were randomly introduced on the lattice space.

Rules that determine the cell growth from one generation to the next in the framework of the territory dynamics were:

### Growth and Cell-Cell Interaction Rules (summer season)

**Figure 1.**
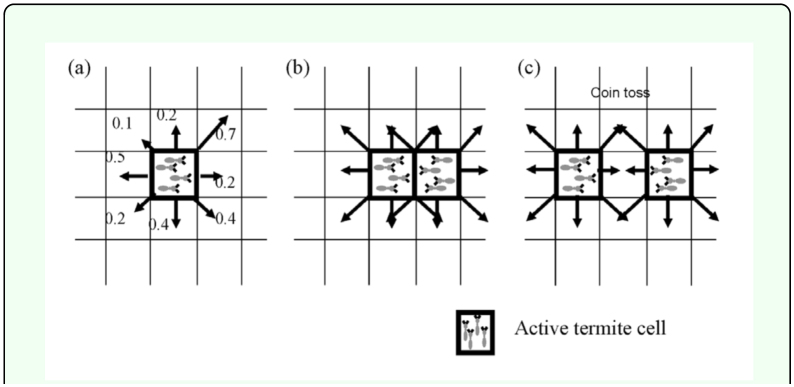
Possible configurations where each active termite cell encounters (a) an empty cell or (bc) another active termite cell. The arrows indicate the direction of growth of each active termite cell and the magnitude of the probability of transition. Squares with termites are active termite cells. High quality figures are available online

- When an active termite cell met empty cells with different values of *P_trans_*, the active termite cell could grow more easily into an empty cell with high *P_tmns_* value than a cell with a low *P_tmns_* value (Figure 1). In Figure 1a, for example, the active termite cells had the highest probability of growing into the top right site.
- When two active termite cells met, they could not share the same site (Figure 1b).
- When more than one active termite cell competed for occupation of an empty site, the occupant of the site was determined by probability of 0.5 (Figure 1c).

### State-Changing Rules (from an active termite cell to an inactive termite cell)

**Figure 2.**
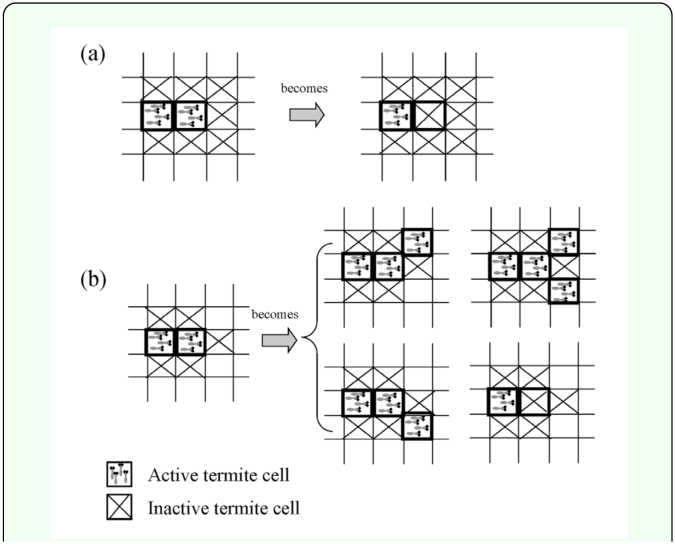
Multiplication process (state-changing rule) of each active termite cell: (a) surrounded by 7 inactive termite cells, or (b) moving towards one of its unoccupied neighbor sites, during I discrete time step t→ *t*+1. High quality figures are available online

- When one active termite cell was surrounded by 7 inactive cells, the active termite cell was changed to an inactive termite cell (Figure 2a) (Su et al. 1991).
- When one active termite cell was surrounded by less than 7 inactive cells, the termite cell could either grow into the empty cells or stop according to the growth rule shown in Figure1a. Once it stopped, it was changed into an inactive termite cell (Figure 2b).

### Shrinkage Rules (winter season)

**Figure 3.**
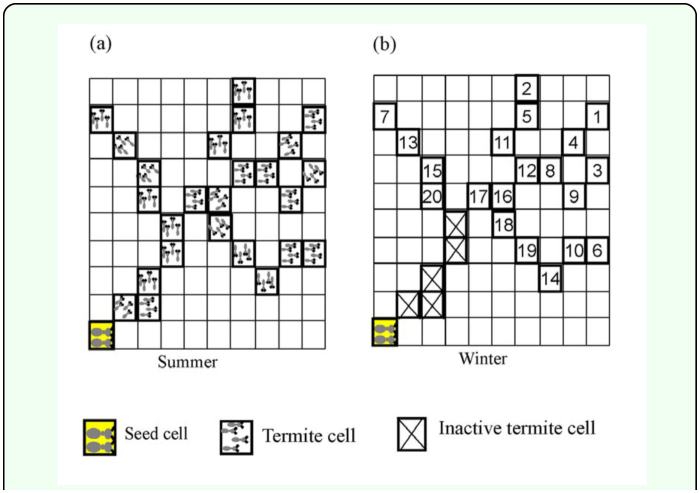
Shrinkage process during winter season. The territory size decreased to ≈ 80%. Termite cells were removed according to their distance from the seed cell. In the right figure, the number in each site represents the changing order from active to inactive termite cell in the shrinkage process. High quality figures are available online

- Messenger and Su (2005) reported that *C. formosanus* colony territories contracted by ∼80% in the winter season, as compared to the summer season. Based on this observation, active/inactive termite cells were removed according to distance. The cells located farthest from the seed cell, which was the active termite cell introduced at the beginning of the simulation run, was removed first; and this process was repeated until percentage of the remaining termite cells, σ, was about 20% (Figure 3).
- After territory size shrank, several of the remaining inactivated cells were stochastically chosen on the basis of their distance from the seed cell; these inactivated cells changed into active termite cells to initiate territory growth (Messenger and Su 2005). The preferential weighting of distal cells is used for determining the starting sites for new tunnel growth stems based on the assumption that growth is most likely formed at the colony's periphery (Lee and Su 2009a).

### Climate Change Rules

- Climate change was characterized by two variables: the cycle duration of the summer season, *T*, and the percentage of the remaining termite cells after the shrinkage during the winter season, σ*.* It has been observed that increases in temperature caused the increase in the value of σ (Messenger and Su 2005). The authors utilized experimental results regarding territory expansion according to temperature by using the underground monitoring stations as described by Su and Scheffrahn (1986). Each station consisted of plastic bucket with a removable lid installed in the soil. Wooden blocks were placed in the bucket. The stations were checked every month to observe if termites were active near the site of station. Consequently, they measured the degree of the termite activation over time and space, which in turn provided some information on the changes in σ according to temperature. For this reason, σ was chosen as a characteristic variable reflecting climate change. The update was synchronous for all cells. The simulation results were statistically averaged over 50 runs.

### Analysis

As the simulation results of this study, we obtained territory size distribution for different *T* and σ, and the slope of the distribution in the size was measured in descending order. In order to differentiate the degree of the slopes, *m*, according to *T* and σ, we clustered data for territory size distribution by using the self-organizing map (SOM). The SOM reduces the dimensions of data through the use of self-organizing neural networks and provides a patterned map of input data without prior knowledge (Kohonen 1988). The patterned nodes are determined according to minimization of distance between input data and weights relating the input and output nodes. In this study *T*, σ, and *m* were used as input vector for training. Initially, the connection weights between output node *j* and input node *i, w_ij_(t)*, were randomly assigned small values. The Euclidian distance at a node *j* on SOM between weight at iteration time *t* and input vector *x* was calculated through learning processes:

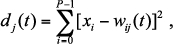
where χ*i* is the value of parameter for the input node *i* (*T* for *i* = 0, σ for *i* = 1, and *m* for *i* = 2), and *P* is the number of the parameter.

The neuron responding maximally to a given input vector was chosen as the winner, the weighted vector of which had the shortest distance to the input vector. For the best matching node and its neighborhood nodes, the new weight vectors were updated as:

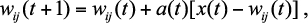
where *t* is the iteration time and α*(t)* is the learning rate. The connection weights changed adaptively at each iteration of the calculation, *t*, until convergence was reached through minimization of the difference, *d_j_(t)*, between input data *x_i_* and the weight *w_ij_(t).* The learning rate accordingly decreased as the system converged. A detailed description regarding application of the SOM is given in Kohonen (1988). After training, the nodes were clustered based on the Ward's linkage method (Dubes 1988). Clustering on by the SOM in behavioural study could be referred to Chon et al. (2004). Training and clustering was conducted by the SOM toolbox in MATLAB (The Mathworks Inc. 2001).

## Results

### Simulation results

**Figure 4.**
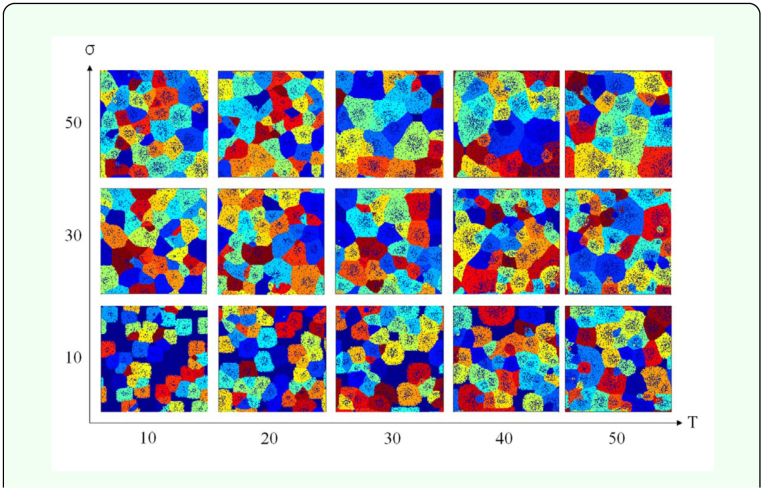
Typical patterns formed at the steady state for *T*=10, 20, 30, 40, and 50, where σ = 10, 30, and 50. Each color indicates each territory. Each territory was defined as the convex polygon containing its corresponding active and inactive termite cells. The black points inside each territory represent empty cells. High quality figures are available online

Figure 4 shows the typical patterns of simulated termite territories at the steady state in the landscape for *N* = 30 under the conditions of *T* = 10, 20, 30, 40, and 50 across σ = 10, 30, and 50. The steady state was reached after sufficiently long time steps. In the state with minimal temperature, territories did not change distinctively along with the time progress across different levels of *T.* As *T* increased, however, variation in territory size was observed. Some territories became larger while others became smaller (Figure 4). This was due to the fact that territories were exposed to a longer time period *T* for competition with their neighbors during the prolonged summer duration, *T.* The competition effect was also demonstrated by the change in σ*.* As σ increased, variation in size was also observed: some territories became larger while others became smaller. A high σ value meant that the amount of territorial shrinkage during winter was small. Thus, when the territories grew after shrinkage, there was a higher chance of bordering with the neighboring territories within a relatively short time. This contributed to increase in competition for space and was comparable to the case of higher values in *T.*

**Figure 5.**
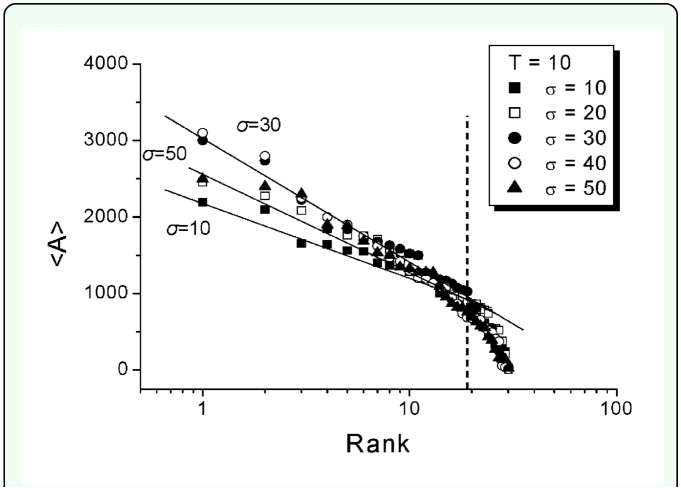
The distribution of average territory sizes, <A>, in descending ranking order for σ = 10, 20, 30, 40, and 50; where *T* = 10. The larger sized territory has the higher rank. High quality figures are available online

In order to measure the effect of climate change on the termite territory, the averaged territory size was investigated, <*A*>, at different combinations of *T* and σ values. Figure 5 shows the territory size distribution in the size descending ranking order at σ = 10, 20, 30, 40, and 50 under the condition of *T* = 10 and *N* = 30. The distribution was separated into two regions according to the rank (indicated as the vertical dotted line) on the *x*-axis: the larger-sized territory was in the front (i.e. the higher rank) and the smaller-sized territory was in the rear (i.e. the lower rank). In this study, our interest was mainly on the front part because the larger-sized territories are likely to mainly reflect the effect of climate change. The smaller-sized territories are governed by the territorial competition in the earlier stage (Lee and Su 2009a,b). The absolute value (*m*) of the slopes in the larger-sized territories in Figure 5 indicates difference in size among territories. The higher value of *m* means that the difference in size among territories is greater. As σ increased, the absolute value of the slope, *m*, was maximized around at σ = 30∼40, but decreased again at the maximal value, σ = 50 (Figure 5). Thus, they reflect little information on the climate effects. It was notable that *m* was less than the maximal value (in absolute value) in comparison with the large value of σ = 50. This result demonstrated that the effect of climate change was maximized within an optimized range of σ values.

**Figure 6.**
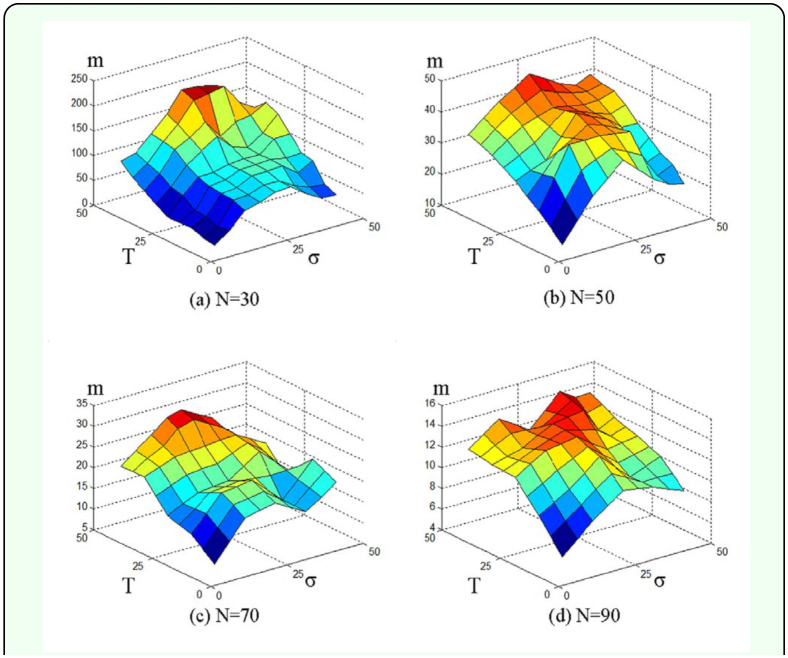
Average m values for 50 simulated territory size distributions for *T* = 10, 15, 20, ... , 50 and σ = 10, 15, 20, ... , 50; where *N* = 30, 50, 70, and 90. High quality figures are available online

In order to better understand the combined effects of *T*, σ, and *N, m* values were computed according to *T* = 10, 15, ... , 50 and σ = 10, 15, ... , 50 in combination across different levels of *N* = 30, 50, 70, and 90 (Figure 6). In general the values of *m* tended to increase with increase in T and σ*.* Regardless of *N*, the values of *m* tended to increase along with the increase in *T.* However, the values were in the maximal range with the optimal level of σ(30 < σ ≤ 40). While the values of σ tended to increase in maximizing *m* at the high levels in T, the degree of optimization appeared to be stronger at low levels in *T.* The values of *m* were consequently observed to be lower in two regions in either maximal or minimal range in σ as shown in all 3-D subfigures in Figure 6.

**Figure 7.**
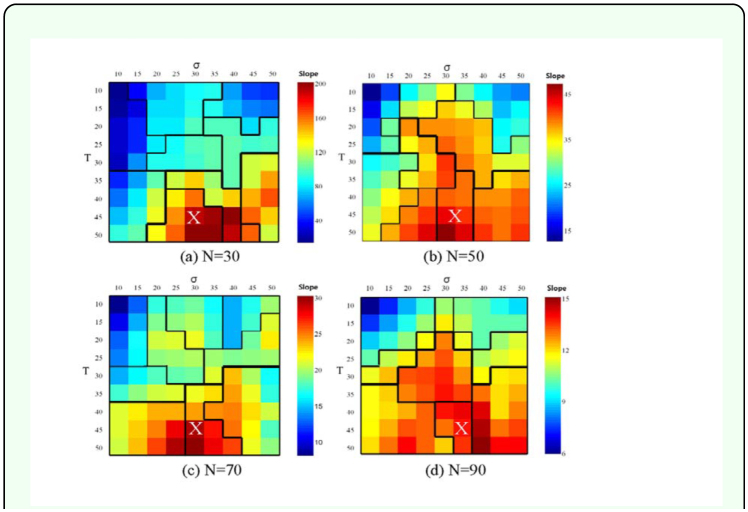
Partitioning of *m* values in relation to σ and *T* according to SOM when *N* = 30, 50, 70, and 90. High quality figures are available online.

Although the gradients were clearly observed, differences in the slopes were not presented. In order to quantitatively verify degree of groupings in the data for the *m* values in different combinations of *T* and σ, we performed SOM (6 × 7 units) to produce clusters of the datasets (i.e. *T, σ* and, and *m*). While the values of *T* and σ are predetermined with the fixed values only *m* values were variable. Grouping of data sets were mainly determined by the closeness in levels of *m* in relative terms with two other input variables. The partitioning of clusters based on the SOM training is presented in Figure 7. Eight clusters were identified on the SOM in each level of *N* = *30*, 50, 70, and 90 according to the Ward's linkage method. The identified clusters for the datasets were correspondingly superposed on the 3-D figures showing the *m* values with different combinations of *T* and σ (Figure 6). The boundaries that separate the groups of *m* values were in accordance with the variation in the *m* values observed in Figure 6. The region for the clusters including the highest range was indicated as “X” in Figure 7. The “X” clusters were separated with other clusters and were correspondingly placed in the area with the maximal *T* values (40 ≤ *T* ≤ 45) and with the intermediate σ values (30 ≤ σ ≤ 40). At *N* = 90 the “X” group did not accommodate the group of the highest values of *m* (σ = 40, and 40 ≤ *T* ≤ 45 in Figure 7d). But these cells still belonged to the neighbor cluster that was most close to the “X” cluster. Within the clusters of “X”, the cells showing some lower levels of *m* were also included. This may be due to nonlinear dimension compression by the SOM training. The groups of lower values of *m* (i.e. two valleys shown in Figure 6) were also correspondingly clustered with boundaries at the upper left and upper right corners in all subfigures in Figure 7.

## Discussion

By using a lattice model similar to the one used by Lee et al. (2007) the effects of climate change on termite's territory size were explored. In the present model, two variables the cycle duration of the summer season, *T*, and the percentage of the remaining termite cell after territory shrinkage, σ — were suitable in presenting territory formation due to climate change. The absolute value of the slope *m*, the slope of the territory size distribution, was varied in accordance with *T* and σ*.* The climate change affected territorial competition through the growth-shrinkage process, which in turn caused changes in territory size distribution (Figure 4). The model was successful in showing both the overall trend of *m* and the maximal ranges in relation to variation in *T* and σ*.* The maximal level of *m* was achieved with the increase in *T* (up to 50), while the maximum level was observed in an optimal range in σ (30 ≤ σ ≤ 40) (Figures 6 and 7).

As explained in Figure 4, high *T* conditions may have caused stronger competition for territories, which gave rise to higher *m* values (i.e. higher differences between territory sizes). The high σ conditions also tended to contribute somewhat to increase in *m.* However, the values of *m* decreased at the maximal values of σ (e.g. *N* = 30 and 70 in Figure 6) under the conditions of high *T* conditions. At both maximal ranges in *T* and σ the colony would experience the strongest competition due to both extended period of activity (*T*) and high chance of bordering (σ). This type of double effects of competition contributed to complexity in differentiating the areas of territories, consequently the size difference somewhat decreased among the territories (i.e. lower slope in absolute value; Figure 5). On the other hand, low *T* and low σ values contributed to weakening the competition due to decrease in the interaction time spent in territories. For low *T* and high σ values, although high σ relatively increased the bordering competition time among territories when territories grew during the next summer season, the low *T* diluted the high σ effect by decreasing the summer period. The results indicated that the effect of climate change accordingly influenced competition under the balance of *T* and σ.

In the course of territory growth, empty sites were formed when a site became fully enclosed by inactive termite cells. The empty cell can become two different states: empty cell and inactive cell. The inactive cells are formed by conformation of surrounding inactive cells. Because the growth of active termite cells into the empty cells is determined by *P_trans_*, the empty cells with lower *P_trans_* can remain without occupation. In this case, neighbor cells of the empty cells may become inactive termite cells according to state changing rule (see Figure 2). Active termite cells located outside the inactive termite cells could not grow into empty sites (see the typical patterns in Figure 4). The density of the empty sites was closely related to territory compactness, which is defined as the ratio of the number of termite cells to the number of empty cells with the same perimeter (Lee et al. 2007). Here, the perimeter means the boundary of the convex polygon: each territory was defined as the convex polygon containing its corresponding active and inactive termite cells. In the present study, however, a direct analysis of territory compactness was excluded in order to stay focused on variation in territory size distribution according to climate change.

The values of the parameters used in this model were based on observational data (Bess 1970; Li et al. 1979; Messenger and Su 2005). However some of the study's assumptions may not be supported in the field. For example, the parameters pertaining to the transition probability might be strongly dependant upon the state of the termites (e.g. age, health, and nutrition) (Lee and Su 2008b, 2009b). Additionally, this model assumed that summer changes into winter in a step-wise fashion. In the field conditions, however, seasonal temperatures fluctuate, and are influenced by a variety of nonlinear factors.

Furthermore, for the purpose of the theoretical simplification we used only two variables, *T* and σ, to characterize the climate change. Other variables such as rainfall patterns and the density of atmospheric carbon dioxide would need to be considered in order to more fully measure the effects of climate change.

Nevertheless, the results of this simulation demonstrated one method of predicting the influence of climatic change on termite territorial behavior. These results will serve as a baseline for future empirical work on the effects of climate change on the foraging territory dynamics of subterranean termites.

## Abbreviation

**SOM**,: self-organizing map
